# The incubation period of Alzheimer’s disease and the timing of tau versus amyloid misfolding and spreading within the brain

**DOI:** 10.1007/s10654-016-0144-8

**Published:** 2016-03-26

**Authors:** Jaap Goudsmit

**Affiliations:** Academic Medical Center of the University of Amsterdam, Amsterdam, The Netherlands; Janssen Prevention Center, Leiden, The Netherlands

Having spent most of my career studying viral infections and their clinical manifestations, in particular AIDS, I have developed a keen interest in the incubation of diseases. In recent years I have focused my attention on Alzheimer’s disease (AD), so for me the key question concerning this illness is: when does AD start to develop? AD is tentatively diagnosed on the basis of a set of clinical symptoms (progressive irreversible decline in memory and other cognitive functions) and confirmed by neuropathology (brain atrophy accompanied by the accumulation of abundant extracellular amyloid β plaques and intraneuronal neurofibrillary tau lesions) [1] (Fig. 1a).

**Fig. 1:**
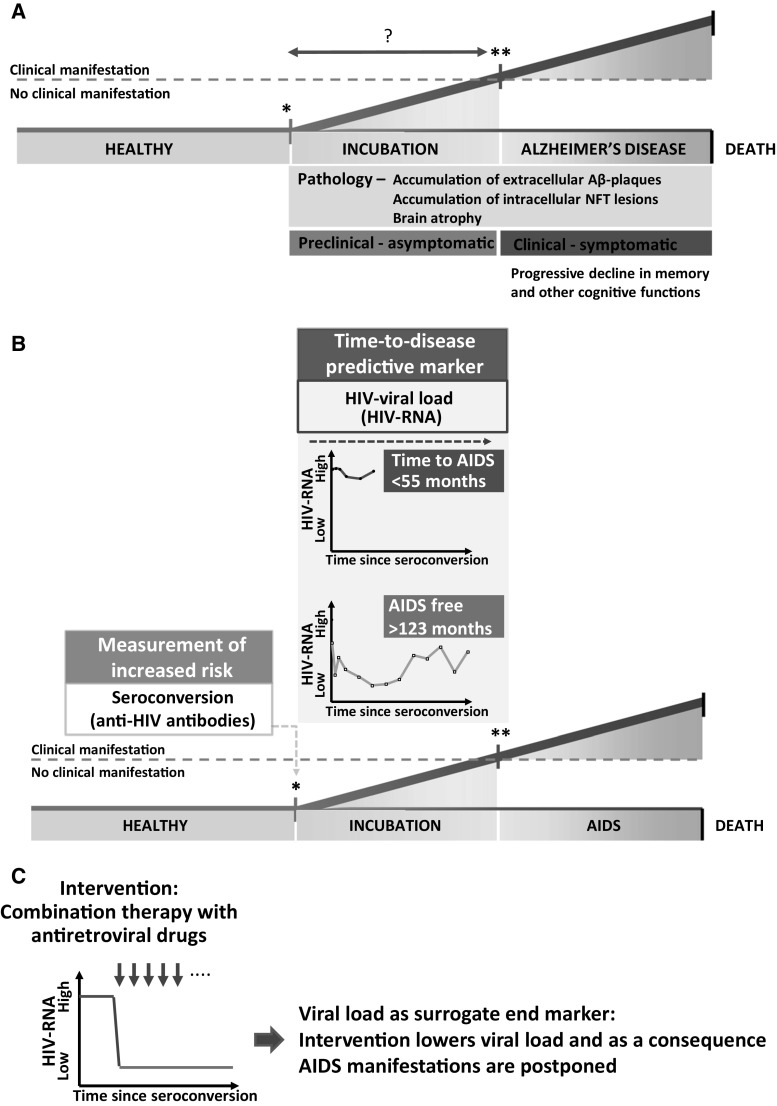
**a** Schematic illustration of the development of Alzheimer’s disease. A ‘healthy’ individual, somewhere in time starts to incubate Alzheimer’s disease, indicated with an *asterisk*. During this incubation time or preclinical asymptomatic phase of the disease, underlying pathology is already ongoing. In the case of AD, this might be 17 years [34]. The initiating event or situation that induces the pathology underlying Alzheimer’s disease (the accumulation of p-tau in the form of neurofibrillary tangles and of amyloid β plaques) is currently not known. When the pathology has increased enough within the incubation time of the disease, clinical symptoms appear, and the patient will be clinically diagnosed as having Alzheimer’s disease. Post-mortem neuropathology will confirm the clinical diagnosis. **b** Diagrams illustrating the use of risk markers, time-to-disease markers and surrogate endpoint markers in the context of preventive interventions against HIV. In the preclinical phase of disease, ‘healthy’ asymptomatic individuals are incubating disease. This incubation time can be short or long. Key for the timely initiation of a preventive intervention is the ability to mark the start (i.e. increased risk) and length (i.e. time to disease) of this incubation time. In the case of HIV, the marker of increased risk is a digital marker, i.e. the presence or absence of anti-HIV antibody positivity. The viral load is a time-to-disease predictive marker. An individual with high HIV viral load develops AIDS in the short term (<55 months) whereas an individual with low HIV viral load will stay AIDS free (Adapted from de Wolf et al. [38]). **c** To determine efficacy of an intervention in the preclinical phase of a disease, surrogate endpoint markers are necessary. In the case of AIDS, the target of the intervention (combination therapy with anti-retroviral drugs) is to lower the viral load and thereby postpone AIDS manifestation. Viral load is thus used as a surrogate endpoint. *NFT* Neurofibrillary tangles, *Aβ* amyloid β

The comparison of AD with AIDS is informative in many ways, even though I do not think that AD is a transmissible or infectious disease [2]. The human retrovirus HIV is both necessary and sufficient to cause AIDS. Following an HIV infection, clinical symptoms defining AIDS can develop within months after infection, but it can also take a decade for AIDS symptoms to appear, depending on the amount of virus continuously produced by CD4^+^ T cells. The number of CD4^+^ T cells decreases due to the HIV infection, and this decrease—to far below the normal physiological threshold—is a hallmark of ongoing HIV infection and continuous production of virus by CD4^+^ T cells. Therefore, the risk of developing AIDS is defined by acquiring the virus in an all-or-nothing manner and the time to disease is defined by the virus load, which may vary from the beginning of infection. Consequently, the level of risk to get AIDS can be measured by a qualitative serum biomarker seroconversion (from antibody negative to positive) and a quantitative serum biomarker increase (from low to high antigen level) [3–11] (Fig. 1b).

Avoiding the (age-independent) risk to acquire HIV prevents AIDS altogether; after the virus has been acquired, combination therapy with antiretroviral drugs reduces the virus load and as a consequence AIDS manifestations are postponed as long as no viral resistance occurs (Fig. 1c). This provides the final proof that HIV causes AIDS.

If we extrapolate these findings to AD, we can ask: will we all get AD if we live long enough? In other words, are we all incubating AD, but are some individuals closer to manifest the disease than others? Or is the risk to develop AD not evenly distributed? Let’s do a thought experiment to examine these questions. Let us start with the idea that AD is a manifestation of biological aging, and that some people may age faster and others slower than the year-by-year progression of calendar age. This can be considered the cause of AD (necessary and sufficient), a significant contributor to the disease (necessary but not sufficient) or a confounder in the classical sense (Fig. 2a). An elegant study by Belsky et al. [12] recently showed that biological age is normally distributed in a cohort of 38-year-olds. While this study included only individuals aged 38 years from the Dunedin Study birth cohort, the biological age of these individuals ranged from 28 to 61 years of age [12]. Biological age was calculated using the Klemera–Doubal algorithm [13] that was validated in the US National Health and Nutrition Survey (NHANES) III dataset [14]. Individuals with an accelerated pace of aging had poorer cognitive function and this difference in cognitive function reflected measurable cognitive decline over the years. Whether this aging effect predicts the eventual development of clinically manifest AD decades later, remains to be established in longitudinal cohort studies (Fig. 2b). If so, do the individuals who age slower or at an average pace in midlife die of old age without AD?

**Fig. 2:**
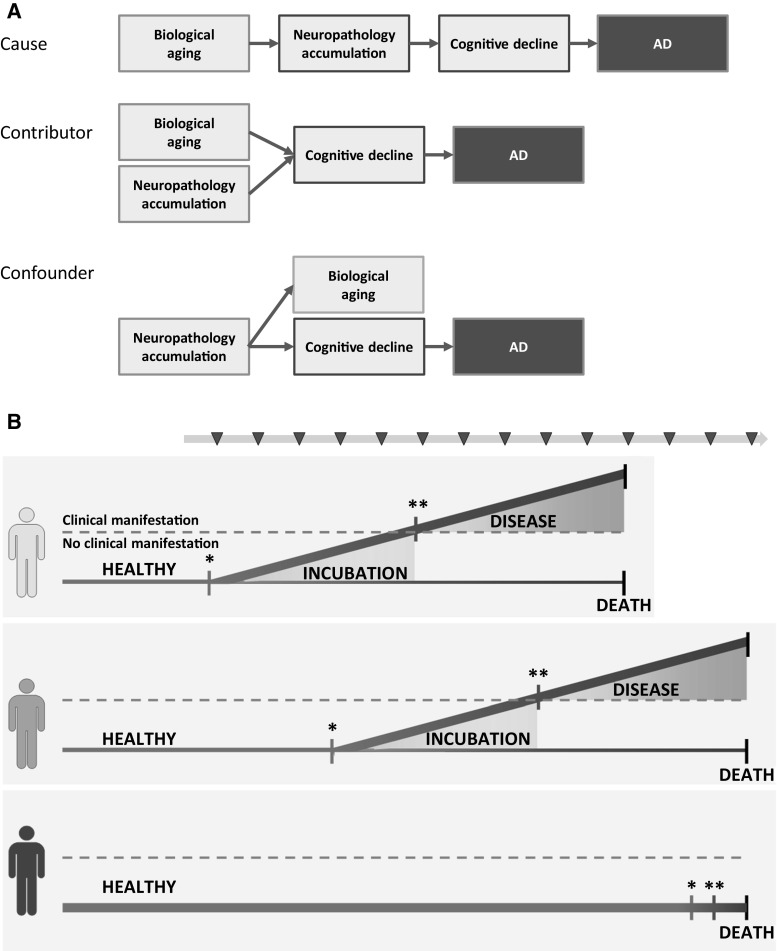
Aging: the cause, a contributor or a confounder of Alzheimer’s disease? **a** Age is the dominant risk factor in Alzheimer’s disease. Recently it was suggested by two groups that accelerated biological aging is associated with cognitive decline [12, 16]. Based on current available data, it cannot be distinguished whether accelerated biological aging is a cause (*upper scenario*), contributor (*mid scenario*) or confounder (*lower scenario*) of Alzheimer’s disease. *Red-framed text-boxes* indicate cause(s). **b** Longitudinal cohort studies are necessary to determine whether biological aging is the cause, a contributor or a confounder of Alzheimer’s disease, and possibly to find other new sensitive and specific predictive markers capable of describing the start and length of the preclinical incubation period of Alzheimer’s disease within an individual. *Start of pathological process; preclinical, asymptomatic phase of AD; **start of clinical, symptomatic AD. *AD* Alzheimer’s disease. *Red arrow* *with series of red triangles* Samples taken during longitudinal study. (Color figure online)

Morgan Levine et al. from the University of California, Los Angeles (UCLA) recently conducted another study of biological brain age, determined not by the Klemera–Doubal algorithm based on simple blood chemistry, viral antibody in serum or plasma and a few clinical tests, but using the so-called ‘epigenetic clock’ designed and built by Steve Horvath of UCLA [15]. The Levine study examined 700 prefrontal cortex samples from Caucasian participants in the Religious Order Study and the Rush Memory and Aging Project, gathering data that suggest that acceleration of biological brain age may be associated with amyloid plaque formation, global cognitive functioning, and episodic memory decline [16]. Levine et al. postulate that aging of the brain may contribute to or even cause the misfolding, aggregation and spreading of both pathological tau (p-tau) and amyloid coinciding with memory loss. Previously, Horvath et al. have shown that blood may be a promising surrogate for brain tissue to determine overall brain age, as the Belsky study independently suggests [12, 17].

It would be extremely helpful if sensitive and specific serum or plasma biomarkers with adequate negative and positive predictive value were available to help establish the risk for AD during the asymptomatic incubation period. This is currently not the case. Although cerebrospinal fluid (CSF) antigen tests for amyloid and tau have been developed (by Innogenetics, Ghent, Belgium; and Thermo Fisher Scientific, Waltham, MA, USA), these tests are impractical for population screening and blood markers are not yet well established [18–21]. The diagnosis of AD can currently be confirmed post-mortem by immunohistochemistry specific for amyloid and tau, and more recently in living individuals using positron emission tomography (PET) scans with ligands for amyloid [22] and/or tau [23, 24].

The most definitive and seminal studies delineating the evolution of tau and amyloid aggregation and spreading have been done by the research group led by Braak [25–27]. Braak et al. [27] conducted immunohistochemistry studies of the brains of individuals who died at age 1–100, using the antibody AT8 to recognize hyperphosphorylated tau in abnormal tau lesions [28] and the antibody 4G8 to identify areas of amyloid accumulation [29, 30] in the brain.

The researchers [27] have shown convincingly that AT8-positive tau deposits, including non-argyrophilic pretangle material, argyrophilic neuropil threads and neurofibrillary tangles (NFT) (Fig. 3a), are present in 100 % of individuals examined from 40 years of age on. While phosphorylation of tau is clearly relevant for AD in the context of its aggregation and toxicity, there is no consensus on the contribution of phosphorylation toward the disease process leading to AD symptoms [31]. It is not clear whether the AT8-immunoreactive pretangle material found, mostly in cases at young age [27], is indeed a forerunner of NFTs or an irrelevant non-specific change in tau. From the age of 40 years on, the percentage of individuals who show spreading of AT8-positive tau to the prefrontal cortex and the neocortex (NFT stages V and VI) is associated most significantly to AD. The increased levels and severity of late-stage hyperphosphorylated tau aggregates appear to be paralleled by a similar process of amyloid assembly, in combination ultimately confirming the clinical diagnosis of AD (Fig. 3b).

**Fig. 3:**
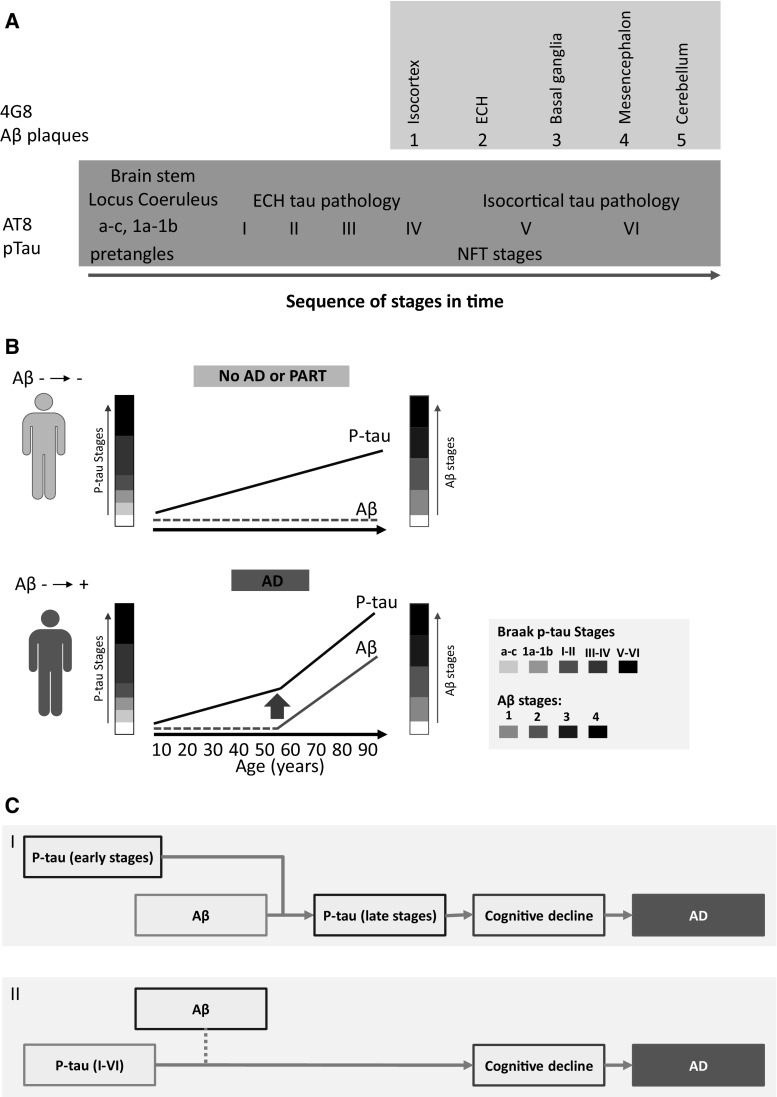
**a** Diagram illustrating the development of amyloid β (*pink box*) and p-tau (*blue box*) pathology (adapted from Duyckaerts et al. [33]). Post-mortem immunohistochemistry evidence of AT8-immunoreactive p-tau material, pretangles and NFT, appears at younger age than 4G8-immunoreactive amyloid β deposits [27]. **b** Diagram illustrating two hypothetical individual situations. The *upper* (*green*) individual does not develop amyloid β or Alzheimer’s disease. Crary et al. suggested diagnosing these cases as PART (primary age-related tauopathy) [32]. The *lower* (*red*) individual does develop amyloid β and AD and shows an acceleration of tauopathy after the appearance of amyloid β [39]. P-tau stages depicted are according to the Braak stages [27]; Amyloid β phases 1–4 are depicted according to the Thal phases of amyloid β plaques [40]. **c** Diagram illustrating the two current main hypotheses of the cause of Alzheimer’s disease. *I* The amyloid hypothesis, which has been the predominant framework for research in Alzheimer’s disease (AD), postulates that amyloid β peptide (Aβ) is the causative agent in AD. It is hypothesized that amyloid β depositions, or possibly amyloid β oligomers, accelerate the already ongoing benign early stages of p-tau pathology towards later stages, eventually resulting in AD [39]. *II* The p-tau hypothesis postulates that p-tau pathology is a continuum of stages of p-tau deposition, which starts early in life with AT8-immunoreactive pretangles and eventually causes AD. Extracellular and aggregated amyloid β depositions may only be produced under pathological conditions by nerve cells that contain abnormal tau (indicated with *dotted blue line*) [36]. *Red-framed text-boxes* indicate cause. (Color figure online)

It is intriguing that half of the 70–80 year olds in this extensive study do not develop 4G8 positive amyloid deposits, despite the presence of AT8-positive pretangle material or NFTs. This observation led some researchers to suggest that a primary tauopathy unrelated to AD exists as a benign age-related entity that might affect all humans if they live long enough [32] (Fig. 3b) and that the clinical diagnosis of AD can only and exclusively be confirmed by the accelerated accumulation of both p-tau and amyloid in the cortex and neocortex. This could mean that p-tau accumulation may be necessary but not sufficient to result in clinical AD or even that AD is solely defined by amyloid plaque formation (Fig. 3c).

So far, there is no evidence to determine whether overt clinical AD occurs in any significant number of individuals who lack amyloid deposition in the brain and what percentage of the population have clinical AD at the time of death with postmortem proof of accumulation of both proteins in the cortex and neocortex. Duyckaerts et al. [33] recently published a paper opposing the concept of a primary age-related tauopathy (PART) proposed by Crary et al. arguing that ‘PART is part of AD’, as defined by low NFT stage (I–III/IV) with no or little amyloid β deposition (Aβ phase 0–2). This debate can only be resolved by evidence from extensive longitudinal cohort studies, which unfortunately are lacking.

If AD starts in young adolescence with the first seeding of p-tau and irreversibly leads to AD eventually, then all humans will get AD depending on their life span, but only if p-tau aggregation, accumulation and spreading are both necessary and sufficient to lead to clinically manifest AD (Fig. 3c). Currently only a minority of the world’s population, albeit accumulating to a very significant number, develops clinical AD. Preventing or removing p-tau aggregation and spreading should on its own be an adequate therapy for AD if p-tau accumulation accounts for the pathogenesis of AD.

Knowing that virtually everybody accumulates p-tau in his or her brain, independently of any clinical signs of AD, and taking into account the relationship between cognitive decline and pace of biological aging, the recently published short-term (3.8 year) longitudinal cohort study among 70-year-olds by Villemagne et al. [34] presents a test case for the idea that amyloid accumulation is in itself sufficient and necessary to develop AD and that there is no such thing as primary age-related amyloidosis (PARA) (Fig. 3c). This study by researchers in the group of Colin Masters (The Florey Institute of Neuroscience and Mental Health, Melbourne, Australia) argues that amyloid β deposition is “neither part of normal aging nor a benign process” and is a prodromal phase of AD, reaching a “threshold of positivity at 17.0 years… before the onset of dementia” [34].

Does AD start with p-tau seeding, aggregation and spreading within the brain at young age [27], or with amyloid β seeding, aggregation and spreading in midlife [35], or with the accelerated combination of the two at older age? Or are tau, amyloid and aging all confounders and is there a yet still unknown cause of AD, such as an environmental factor [36, 37]. Finding the answer will depend on longitudinal natural history studies evaluating the incubation and prodromes of AD as well as its clinical progression of AD, in conjunction with PET scans done at regular intervals using p-tau and amyloid ligands. Only then can we answer the question of what induces the seeding of either p-tau or amyloid. Only then can non-invasive biomarker assays be qualified and validated.

How will we unravel the pathogenesis underlying AD? I believe the way forward lies with research into specific interventions using small molecules specifically interfering with the overproduction of p-tau or amyloid, and/or antibodies blocking the seeding, aggregation or spreading of p-tau and/or amyloid. Observing the effects of these interventions, given at distinct early time points in the course of AD development, on both clinical endpoints and quantitative PET scans might be the route to finally resolving the question of what causes AD.

## References

[1] Goedert M (2015). Neurodegeneration. Alzheimer’s and Parkinson’s diseases: the prion concept in relation to assembled Abeta, tau, and alpha-synuclein. Science.

[2] Goudsmit J, Morrow CH, Asher DM (1980). Evidence for and against the transmissibility of Alzheimer disease. Neurology.

[3] de Wolf F, Goudsmit J, Paul DA (1987). Risk of AIDS related complex and AIDS in homosexual men with persistent HIV antigenaemia. Br Med J (Clin Res Ed).

[4] de Wolf F, Lange JM, Houweling JT (1989). Appearance of predictors of disease progression in relation to the development of AIDS. AIDS.

[5] Goudsmit J, de Wolf F, Paul DA (1986). Expression of human immunodeficiency virus antigen (HIV-Ag) in serum and cerebrospinal fluid during acute and chronic infection. Lancet.

[6] Goudsmit J, Lange JM, Paul DA, Dawson GJ (1987). Antigenemia and antibody titers to core and envelope antigens in AIDS, AIDS-related complex, and subclinical human immunodeficiency virus infection. J Infect Dis.

[7] Goudsmit J, Wolters EC, Bakker M (1986). Intrathecal synthesis of antibodies to HTLV-III in patients without AIDS or AIDS related complex. Br Med J (Clin Res Ed).

[8] Lange J, Goudsmit J (1987). Decline of antibody reactivity to HIV core protein secondary to increased production of HIV antigen. Lancet.

[9] Lange JM, Coutinho RA, Krone WJ (1986). Distinct IgG recognition patterns during progression of subclinical and clinical infection with lymphadenopathy associated virus/human T lymphotropic virus. Br Med J (Clin Res Ed).

[10] Lange JM, Paul DA, Huisman HG (1986). Persistent HIV antigenaemia and decline of HIV core antibodies associated with transition to AIDS. Br Med J (Clin Res Ed).

[11] Goudsmit J, Epstein LG, Paul DA (1987). Intra-blood-brain barrier synthesis of human immunodeficiency virus antigen and antibody in humans and chimpanzees. Proc Natl Acad Sci USA.

[12] Belsky DW, Caspi A, Houts R (2015). Quantification of biological aging in young adults. Proc Natl Acad Sci USA.

[13] Klemera P, Doubal S (2006). A new approach to the concept and computation of biological age. Mech Ageing Dev.

[14] Levine ME (2013). Modeling the rate of senescence: can estimated biological age predict mortality more accurately than chronological age?. J Gerontol A Biol Sci Med Sci.

[15] Horvath S (2013). DNA methylation age of human tissues and cell types. Genome Biol.

[16] Levine ME, Lu AT, Bennett DA, Horvath S (2015). Epigenetic age of the pre-frontal cortex is associated with neuritic plaques, amyloid load, and Alzheimer’s disease related cognitive functioning. Aging (Albany NY).

[17] Horvath S, Zhang Y, Langfelder P (2012). Aging effects on DNA methylation modules in human brain and blood tissue. Genome Biol.

[18] Zetterberg H, Wilson D, Andreasson U (2013). Plasma tau levels in Alzheimer’s disease. Alzheimers Res Ther.

[19] Fiandaca MS, Kapogiannis D, Mapstone M (2015). Identification of preclinical Alzheimer’s disease by a profile of pathogenic proteins in neurally derived blood exosomes: a case-control study. Alzheimers Dement.

[20] Wang T, Xiao S, Liu Y (2014). The efficacy of plasma biomarkers in early diagnosis of Alzheimer’s disease. Int J Geriatr Psychiatry.

[21] Ingelson M, Blomberg M, Benedikz E (1999). Tau immunoreactivity detected in human plasma, but no obvious increase in dementia. Dement Geriatr Cogn Disord.

[22] Klunk WE, Engler H, Nordberg A (2004). Imaging brain amyloid in Alzheimer’s disease with Pittsburgh Compound-B. Ann Neurol.

[23] Chien DT, Bahri S, Szardenings AK (2013). Early clinical PET imaging results with the novel PHF-tau radioligand [F-18]-T807. J Alzheimers Dis.

[24] Chien DT, Szardenings AK, Bahri S (2014). Early clinical PET imaging results with the novel PHF-tau radioligand [F18]-T808. J Alzheimers Dis.

[25] Braak H, Alafuzoff I, Arzberger T, Kretzschmar H, Del Tredici K (2006). Staging of Alzheimer disease-associated neurofibrillary pathology using paraffin sections and immunocytochemistry. Acta Neuropathol.

[26] Braak H, Braak E (1991). Neuropathological stageing of Alzheimer-related changes. Acta Neuropathol.

[27] Braak H, Thal DR, Ghebremedhin E, Del Tredici K (2011). Stages of the pathologic process in Alzheimer disease: age categories from 1 to 100 years. J Neuropathol Exp Neurol.

[28] Mercken M, Vandermeeren M, Lubke U (1992). Monoclonal antibodies with selective specificity for Alzheimer Tau are directed against phosphatase-sensitive epitopes. Acta Neuropathol.

[29] Kim KS, Miller DL, Sapienza VJ, Chen CJ, Bai C, Grunke-Iqbal I, Currie JR, Wisniewski HM (1988). Production and characterization of monoclonal antibodies reactive to synthetic cerebrovascular amyloid peptide. Neurosci Res Commun.

[30] Hatami A, Monjazeb S, Glabe C (2015). The anti-amyloid-beta monoclonal antibody 4G8 recognizes a generic sequence-independent epitope associated with alpha-Synuclein and Islet amyloid polypeptide amyloid fibrils. J Alzheimers Dis.

[31] Tenreiro S, Eckermann K, Outeiro TF (2014). Protein phosphorylation in neurodegeneration: friend or foe?. Front Mol Neurosci.

[32] Crary JF, Trojanowski JQ, Schneider JA (2014). Primary age-related tauopathy (PART): a common pathology associated with human aging. Acta Neuropathol.

[33] Duyckaerts C, Braak H, Brion JP (2015). PART is part of Alzheimer disease. Acta Neuropathol.

[34] Villemagne VL, Burnham S, Bourgeat P (2013). Amyloid beta deposition, neurodegeneration, and cognitive decline in sporadic Alzheimer’s disease: a prospective cohort study. Lancet Neurol.

[35] Jack CR, Knopman DS, Jagust WJ (2013). Tracking pathophysiological processes in Alzheimer’s disease: an updated hypothetical model of dynamic biomarkers. Lancet Neurol.

[36] Braak H, Del Tredici K (2015). The preclinical phase of the pathological process underlying sporadic Alzheimer’s disease. Brain.

[37] Itzhaki RF (2014). Herpes simplex virus type 1 and Alzheimer’s disease: increasing evidence for a major role of the virus. Front Aging Neurosci.

[38] de Wolf F, Spijkerman I, Schellekens PT (1997). AIDS prognosis based on HIV-1 RNA, CD4+ T-cell count and function: markers with reciprocal predictive value over time after seroconversion. AIDS.

[39] Musiek ES, Holtzman DM (2015). Three dimensions of the amyloid hypothesis: time, space and ‘wingmen’. Nat Neurosci.

[40] Thal DR, Rub U, Schultz C (2000). Sequence of Abeta-protein deposition in the human medial temporal lobe. J Neuropathol Exp Neurol.

